# Signal Integration in Quorum Sensing Enables Cross-Species Induction of Virulence in *Pectobacterium wasabiae*

**DOI:** 10.1128/mBio.00398-17

**Published:** 2017-05-23

**Authors:** Rita S. Valente, Pol Nadal-Jimenez, André F. P. Carvalho, Filipe J. D. Vieira, Karina B. Xavier

**Affiliations:** Instituto Gulbenkian de Ciência, Oeiras, Portugal; Princeton University

## Abstract

Bacterial communities can sense their neighbors, regulating group behaviors in response to cell density and environmental changes. The diversity of signaling networks in a single species has been postulated to allow custom responses to different stimuli; however, little is known about how multiple signals are integrated and the implications of this integration in different ecological contexts. In the plant pathogen *Pectobacterium wasabiae* (formerly *Erwinia carotovora*), two signaling networks—the N-acyl homoserine lactone (AHL) quorum-sensing system and the Gac/Rsm signal transduction pathway—control the expression of secreted plant cell wall-degrading enzymes, its major virulence determinants. We show that the AHL system controls the Gac/Rsm system by affecting the expression of the regulatory RNA RsmB. This regulation is mediated by ExpR2, the quorum-sensing receptor that responds to the *P. wasabiae* cognate AHL but also to AHLs produced by other bacterial species. As a consequence, this level of regulation allows *P. wasabiae* to bypass the Gac-dependent regulation of RsmB in the presence of exogenous AHLs or AHL-producing bacteria. We provide *in vivo* evidence that this pivotal role of RsmB in signal transduction is important for the ability of *P. wasabiae* to induce virulence in response to other AHL-producing bacteria in multispecies plant lesions. Our results suggest that the signaling architecture in *P. wasabiae* was coopted to prime the bacteria to eavesdrop on other bacteria and quickly join the efforts of other species, which are already exploiting host resources.

## IMPORTANCE

Quorum-sensing mechanisms enable bacteria to communicate through small signal molecules and coordinate group behaviors. Often, bacteria have various quorum-sensing receptors and integrate information with other signal transduction pathways, presumably allowing them to respond to different ecological contexts. The plant pathogen *Pectobacterium wasabiae* has two N-acyl homoserine lactone receptors with apparently the same regulatory functions. Our work revealed that the receptor with the broadest signal specificity is also responsible for establishing the link between the main signaling pathways regulating virulence in *P. wasabiae*. This link is essential to provide *P. wasabiae* with the ability to induce virulence earlier in response to higher densities of other bacterial species. We further present *in vivo* evidence that this novel regulatory link enables *P. wasabiae* to join related bacteria in the effort to degrade host tissue in multispecies plant lesions. Our work provides support for the hypothesis that interspecies interactions are among the major factors influencing the network architectures observed in bacterial quorum-sensing pathways.

## INTRODUCTION

**FIG 1:**
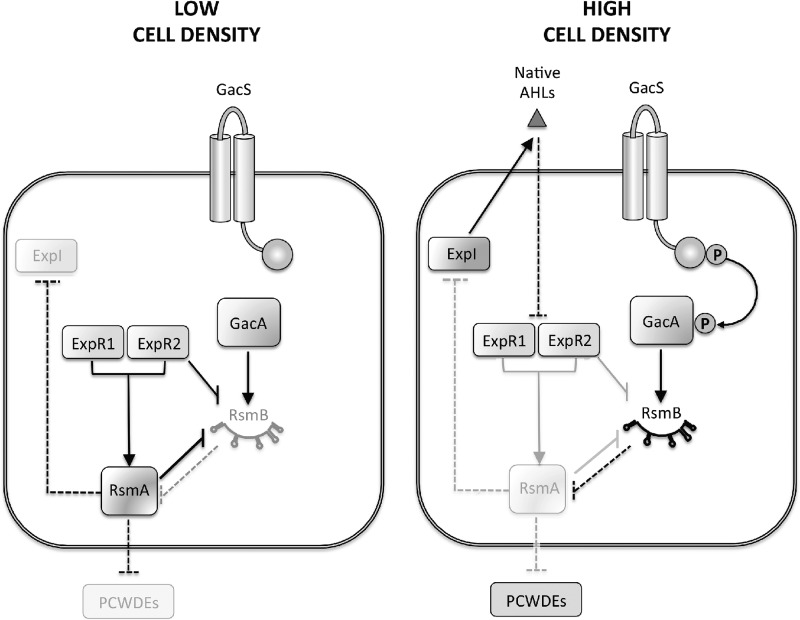
Major signal transduction pathways regulating PCWDEs in *Pectobacterium* spp. In *Pectobacterium* spp. at low cell density, when the AHL signals are low, ExpR1 and ExpR2 induce the transcription of *rsmA*, repressing expression of PCWDEs and the AHL synthase ExpI. As cell density increases, AHLs accumulate and inactivate ExpR1 and ExpR2. As a result, *rsmA* transcription is no longer induced. The GacS/GacA system is also active at high cell density and promotes the transcription of *rsmB*, a noncoding RNA that blocks RsmA activity by sequestration. Inhibition of RsmA will, in turn, result in increased production of PCWDEs and induction of virulence. We show here that regulation of RsmB is not exclusively controlled by the GacS/GacA system but is also regulated by AHLs. In the absence of AHLs, ExpR1 and ExpR2 repress the expression of *rsmB*. Repression of *rsmB* mediated by ExpR1 requires RsmA, while ExpR2 represses RsmB in an RsmA-dependent and -independent manner. Therefore, at high cell density, *rsmB* expression increases as a result of the activation mediated by the GacS/GacA system and relief of the repression mediated by ExpR1 and ExpR2. Gray and black lines/boxes indicate inactive and active pathways, respectively. Arrows indicate activation, while intersecting lines indicate repression. Solid lines indicate transcriptional regulation, while dashed lines indicate posttranscriptional and posttranslational mechanisms. Not all of the regulatory interactions displayed here have been proven to occur through direct interactions.

Bacteria use different signaling systems to communicate with one another, synchronizing gene expression and coordinating their functions to engage in group behaviors (1, 2). However, the mechanisms by which cells integrate and interpret multiple signals received by their neighbors and the surrounding environment, as well as the consequences of such integration, remain largely unknown. The *Pectobacterium* genus includes an important group of plant pathogens whose virulence is characterized by the production of a large array of pectolytic plant cell wall-degrading enzymes (PCWDEs). Two main signaling pathways are responsible for the coordinated production of these virulence factors: the quorum-sensing N-acyl homoserine lactone (AHL) system and the Gac/Rsm system (Fig. 1). These two signal transduction pathways are also used by multiple gammaproteobacteria to regulate virulence (3–8).

In *Pectobacterium wasabiae*, the AHL system includes ExpI, a LuxI-type AHL synthase, and two AHL receptors, ExpR1 and ExpR2, which are LuxR homologues. These two receptors control the transcription of *rsmA*, a global repressor of virulence (9). Unlike in most AHL receptors, the DNA binding activity of ExpR1 and ExpR2 is repressed by ligand binding, and thus, AHLs prevent the transcription of *rsmA*, derepressing the expression of virulence mainly through the control of PCWDE production. These two quorum-sensing receptors have different ligand specificities: ExpR1 recognizes the main AHL produced by *P. wasabiae* (3-oxo-C8-HSL, 3-oxo-octanoyl homoserine lactone) and to a lesser extent 3-oxo-C10-HSL (3-oxo-decanoyl homoserine lactone), ExpR2 binds to a broader range of AHLs, such as C6-HSL (hexanoyl homoserine lactone), 3-oxo-C6-HSL, C7-HSL (heptanoyl homoserine lactone), 3-oxo-C8-HSL, and, to a lesser extent, C8-HSL (octanoyl homoserine lactone) and 3-oxo-C10-HSL (decanoyl homoserine lactone) (9–12). Some of these AHLs are produced by other pectolytic bacteria, while *P. wasabiae* produces mainly 3-oxo-C8-HSL and trace amounts of C8-HSL and oxo-C6-HSL (9). Therefore, it has been proposed that ExpR1 responds mainly to the cognate signal (3-oxo-C8-HSL) while ExpR2 responds to the cognate signal but also to AHLs produced by other species (11).

The Gac/Rsm system comprises the two-component GacS/GacA system (also named ExpS/ExpA in *Pectobacterium* species) and the noncoding regulatory RNA RsmB. Upon activation, GacS phosphorylates the transcriptional regulator GacA, which, in turn, activates the transcription of *rsmB*. RsmB binds to RsmA, neutralizing its mRNA binding activity (13, 14) and derepressing the translation of all RsmA targets, including that of PCWDEs (11, 15, 16).

Despite many efforts to identify the signal(s) responsible for the activation of the Gac/Rsm system, the precise chemical structure of this signal(s) remains elusive (17). Accumulation of intermediates of the Krebs cycle and production of short-chain fatty acids at low pH have been shown to stimulate the system. However, the relevant stimuli leading to the accumulation of these metabolites and activation of the system at neutral pH have not been identified (18–20). Recently, extracellular potassium and calcium have been described as important environmental signals that trigger activation of the Gac/Rsm system in *P. wasabiae* and *Pseudomonas aeruginosa*, respectively (21, 22). Nonetheless, other signals, presumably self-produced molecules, that accumulate in the extracellular growth medium are still required for full activation (23).

In *Pectobacterium* spp., the *rsmA* promoter is the only known direct target of ExpR1 and ExpR2 (10, 11, 24). At low cell density, the absence of AHLs results in ExpR1 and ExpR2 binding to the *rsmA* promoter region, activating its transcription and repressing virulence, while at high cell density, high AHL concentrations result in inhibition of the binding of ExpR1 and ExpR2 to the *rsmA* promoter, preventing *rsmA* transcription. Repression of RsmA expression by AHLs together with its sequestration by RsmB, results in inhibition of RsmA-mediated regulation and consequent virulence expression (10, 11, 24). Thus, in *Pectobacterium* spp., both pathways converge at the level of RsmA: while the AHL system controls *rsmA* transcription (11, 25), Gac/Rsm posttranscriptionally regulates RsmA activity via the binding of RsmB to RsmA (14), (Fig. 1). Additionally, there is evidence of feedback inhibition mediated by RsmA where, at low cell density, when RsmA levels are high, the mRNA of *expI* is targeted by RsmA, decreasing ExpI production (15).

Here we investigated the mechanisms of signal integration in *P. wasabiae*, a bacterium known to rely on the integration of multiple signals to regulate virulence expression and assess population density (3). We report that the AHL network also controls the Gac/Rsm system by modulating the transcription of *rsmB*. We show that this novel level of regulation is essential for the ability of *P. wasabiae* to respond to the presence of other AHL-producing bacteria in multispecies plant lesions. By studying the architecture of the major signaling networks regulating virulence in *P. wasabiae*, we provide *in vivo* evidence that integration of multiple signaling pathways can be important in the regulation of bacterial group behaviors in multispecies communities.

## RESULTS

### Two signaling pathways are required to control virulence in *P. wasabiae.*

**FIG 2:**
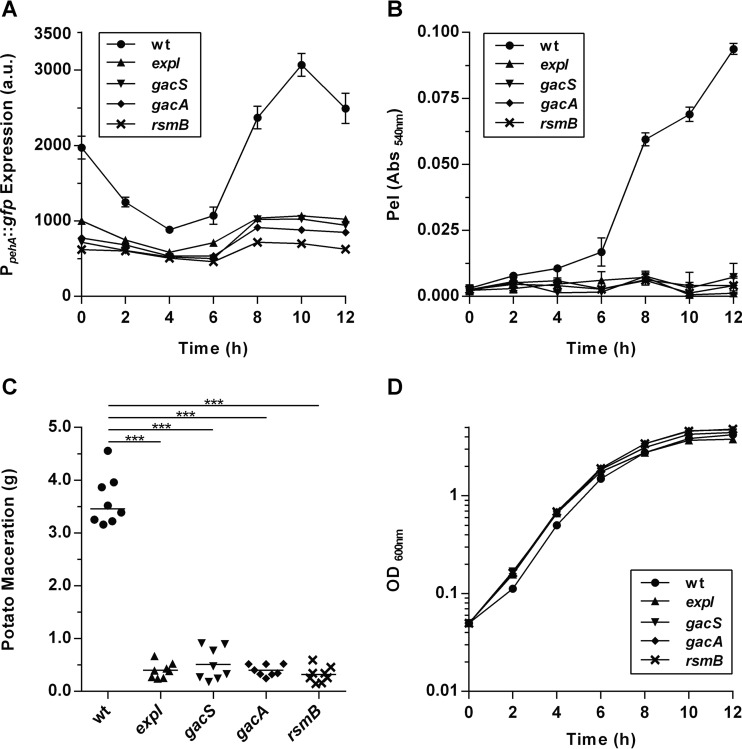
Both *expI* and the Gac/Rsm system are required for virulence in *P. wasabiae*. Virulence expression in WT *P. wasabiae* SCC3193 and *expI*, *gacS*, *gacA*, and *rsmB* mutants was measured with a P_*pehA*_::*gfp* reporter fusion (A), the Pel enzymatic assay (B), and a potato infection assay at 48 h (C). Growth curves of the strains used are shown in panel D. ***, *P* < 0.001 by Mann-Whitney test (*n* = 8). Holm-Bonferroni correction was used for multiple comparisons. a.u., arbitrary units. Error bars represent the standard deviation of the mean. For panels A, B, and D, *n* = 3. Abs, absorbance.

Previous genetic studies targeting genes affecting virulence and PCWDE production in *Pectobacterium* spp. identified transposon insertions in *expI*, *gacS*, and *gacA* (13, 26, 27). However, while a *gacA*::Tn*10* mutant was completely avirulent, a *gacS*::Tn*10* mutant was partially attenuated. We determined the virulence phenotype in *gacA* and *gacS* full-deletion mutants of *P. wasabiae* SCC3193 (28). Polygalacturonase and pectate lyase are two of the main PCWDEs in *Pectobacterium* spp. Analysis of *pehA* (polygalacturonase gene) promoter expression (Fig. 2A), enzymatic activity of the pectate lyase (Pel) (Fig. 2B), and virulence in a potato maceration infection model in *P. wasabiae* revealed that virulence was fully abolished in both the *gacA* and *gacS* deletion mutants (Fig. 2C). Supporting the possibility that the transposon insertion in the *gacS*::Tn*10* mutant described previously is not a full loss-of-function mutant (27). We have also constructed a mutant with full deletion of *rsmB*, the major target of the GacS/GacA two-component system, and evaluated its virulence. The phenotype of the *rsmB* deletion mutant was the same as that of the *gacA* and *gacS* mutants (Fig. 2). None of these mutants had growth defects, as shown by the growth curves in Fig. 2D. Additionally, in the *expI* mutant that had been described as being fully avirulent (26), expression of *pehA*, Pel activity, and virulence were as low as in all of the Gac/Rsm deletion mutants (Fig. 2). These results demonstrated that both signaling pathways are required to activate virulence and are dependent on one another. Therefore, to understand the molecular basis of this observation, we decided to investigate the existence of regulatory links between the two pathways. As ExpI is a target of RsmA (15), we hypothesized that the GacS/GacA system could control AHL levels. We measured AHL levels in the different Gac/Rsm pathway mutants, confirming lower production in the *gacS*, *gacA*, and *rsmB* mutants (see Fig. S1 in the supplemental material). These results provided an explanation of how the AHL quorum-sensing network requires the Gac/Rsm pathway to activate virulence. Next, we investigated whether the AHL network could also regulate the Gac/Rsm pathway, since such a link between the two pathways could explain the avirulence phenotype observed in an *expI* mutant.

10.1128/mBio.00398-17.1 FIG S1  AHL concentrations of WT *P. wasabiae* and *expI*, *gacS*, *gacA*, and *rsmB* mutants measured at different time points. To quantify the extracellular concentration of AHLs (A), overnight cultures were diluted to an OD_600_ of 0.05 in LB broth plus PGA and incubated at 30°C with aeration. At the times indicated, culture supernatants were collected with a 96-well filter plate (Millipore MAGVS2210 MultiScreen) and the concentrations of AHLs within these supernatants were measured as follows. *E. coli* strain JM109 containing the pSB401 reporter (1) was grown overnight at 37°C in LB broth plus tetracycline and diluted 1:100 in fresh medium. A 1 mM stock solution of 3-oxo-C8-HSL (Sigma O1764) was successively diluted from 2 to 0.0625 μM and used as a calibration curve to quantify the concentrations of AHLs in the cell-free culture fluids. For each sample, 30 μl of cell-free culture fluid or AHL preparation was incubated with 170 μl of the diluted reporter strain at 37°C with aeration for 4 h. Luminescence (which is induced proportionally to the AHL concentration) was measured with a Victor^3^ multilabel counter (PerkinElmer). The growth curves of the strains used are shown in panel B. Error bars represent the standard deviation of the mean. *n* = 3. See Text S1 for references. Download FIG S1, PDF file, 0.3 MB. Copyright © 2017 Valente et al. 2017 Valente et al. This content is distributed under the terms of the Creative Commons Attribution 4.0 International license.

10.1128/mBio.00398-17.6 TEXT S1  Supplement Bibliography. Download TEXT S1, PDF file, 0.4 MB. Copyright © 2017 Valente et al. 2017 Valente et al. This content is distributed under the terms of the Creative Commons Attribution 4.0 International license.

### AHLs control RsmB, the major target of the GacS/GacA two-component system.

**FIG 3:**
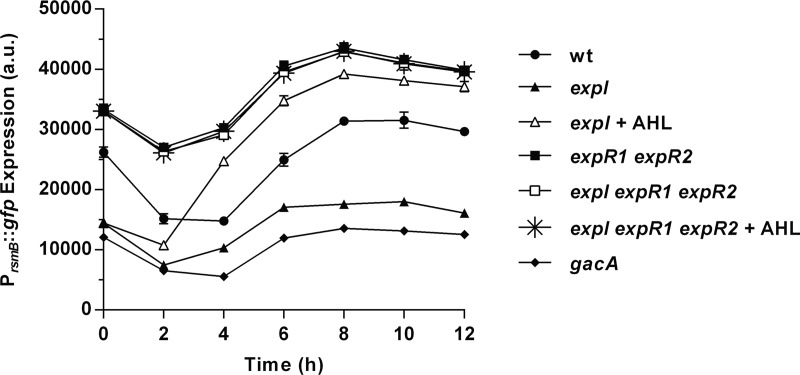
Expression of *rsmB* in *P. wasabiae* is regulated by AHLs. P_*rsmB*_::*gfp* expression was measured in WT *P. wasabiae* SCC3193 and *expI*, *expR1 expR2*, *expI expR1 expR2*, and *gacA* mutants. Complementation with AHLs was performed with 2 µM 3-oxo-C8-HSL. a.u., arbitrary units. Error bars represent the standard deviation of the mean. *n* = 3.

**FIG 4:**
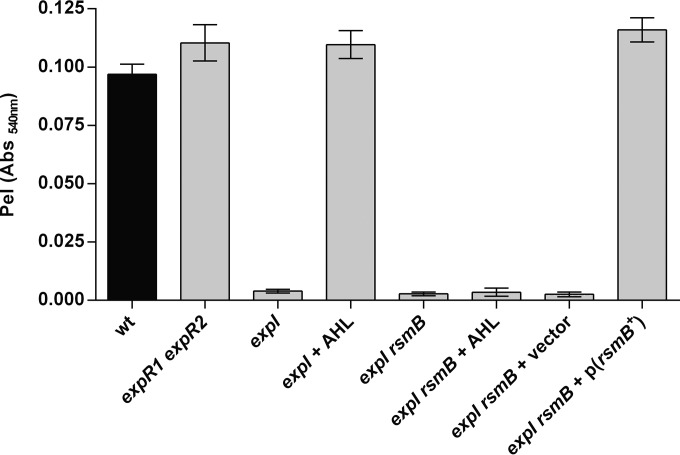
Regulation of Pel activity by AHLs requires RsmB. Pel enzymatic assay was measured in WT *P. wasabiae* SCC3193 and *expR1 expR2*, *expI*, and *expI rsmB* mutants. Complementation with AHLs was performed with 2 µM 3-oxo-C8-HSL. *rsmB* expression was induced with 1 µM IPTG. Cells were collected when cultures reached an OD_600_ of approximately 3.5. Error bars represent the standard deviation of the mean. *n* = 7. Abs, absorbance.

A clue to a possible link between the AHL and the Gac/Rsm pathway came from a genetic screening performed in our laboratory that revealed *expI* as a potential regulator of RsmB (21). To explore this possibility, we analyzed *rsmB* transcription in wild-type (WT) and AHL quorum-sensing pathway mutants in the background of the *P. wasabiae* SCC3193 strain, both in the absence and presence of exogenous 3-oxo-C8-HSL (the major AHL produced by *P. wasabiae*). As shown in Fig. 3, WT *P. wasabiae* regulates *rsmB* transcription in a cell density-dependent manner. At early stages of growth, the transcription of *rsmB*, measured by *gfp* expression in WT cells carrying a P_*rsmB*_::*gfp* promoter fusion, is turned off and is activated only after 6 h of growth as cells reach high density (optical density at 600 nm [OD_600_] of >1) (circles, Fig. 2D and 3). In contrast, in the *expI* mutant, expression was much lower than in the WT at all stages of growth and only slightly higher than in the *gacA* mutant, which is thought to be the main activator of *rsmB* (closed triangles and diamonds, respectively, Fig. 2D and 3). The transcription of *rsmB* in the *expI* mutant could be induced by exogenous addition of 3-oxo-C8-HSL to the growth medium (open triangles, Fig. 3). This AHL-dependent regulation relies on the known AHL receptors (ExpR1 and ExpR2), as shown by the high levels of *rsmB* transcription and the lack of response to 3-oxo-C8-HSL observed in the *expI expR1 expR2* triple mutant (Fig. 3). We next determined if this AHL-dependent regulation of *rsmB* could influence virulence factor production. In the *expI* mutant, Pel activity is low, even in cultures grown to a high cell density (Fig. 4). But as expected, addition of AHLs can restore induction of the production of these enzymes to WT levels, as observed by the levels of Pel activity obtained in the *expI* mutant cultured in the presence of exogenously supplied 3-oxo-C8-HSL (Fig. 4). Accordingly, Pel activity was also derepressed in an *expR1 expR2* mutant. In contrast, in the absence of *rsmB*, addition of AHLs could not restore Pel activity, as demonstrated by the addition of 3-oxo-C8-HSL to the *expI rsmB* double mutant. Additionally, overexpression of *rsmB* under the control of a *lac* promoter in an *expI* mutant, in the absence of AHLs, was sufficient to fully rescue Pel activity (Fig. 4). These results demonstrate that AHLs regulate *rsmB* and that this regulation is essential for the AHL-dependent control of virulence.

### ExpR2 is responsible for the RsmA-independent control of RsmB.

**FIG 5:**
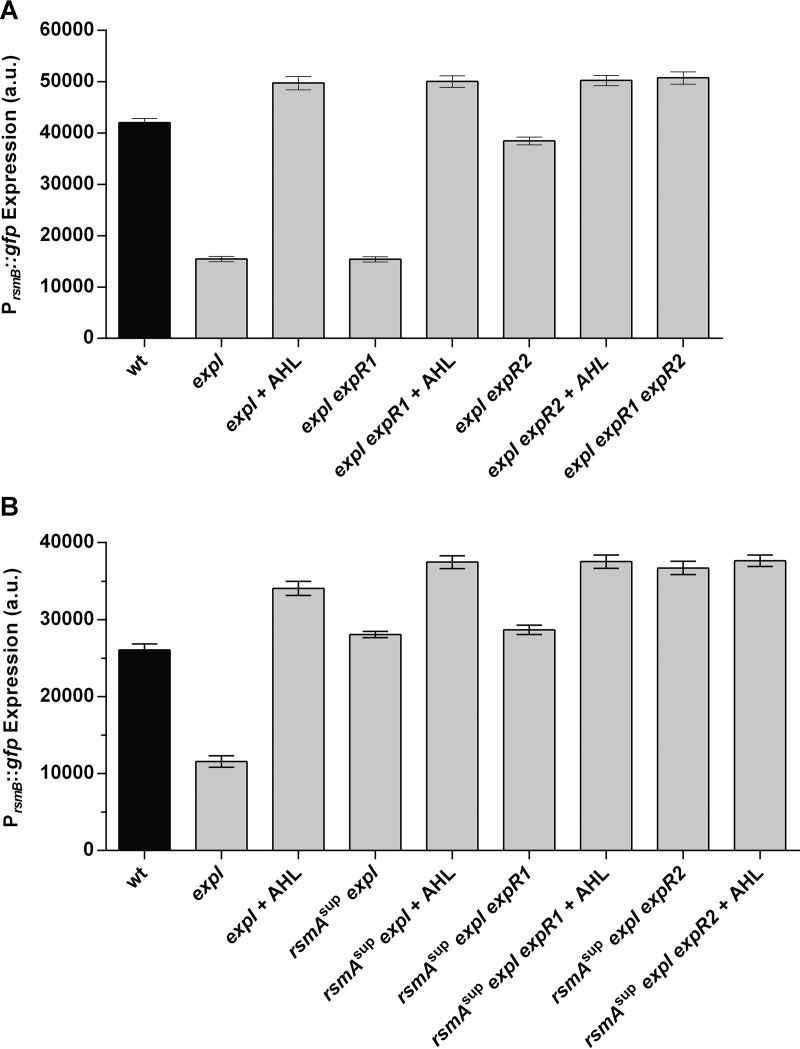
Expression of *rsmB* is controlled by ExpR2 in an RsmA-independent manner. Comparison of the effects of AHLs on P_*rsmB*_::*gfp* expression in the presence (A) or absence (B) of RsmA. Complementation with AHLs was performed with 2 µM 3-oxo-C8-HSL. Cells were collected when cultures reached an OD_600_ of approximately 3.5. a.u., arbitrary units. Error bars represent the standard deviation of the mean. *n* = 7.

We next determined the different contributions of ExpR1 and ExpR2 to the AHL-dependent regulation of *rsmB* transcription. As shown in Fig. 5A, both ExpR1 and ExpR2 regulate the AHL-dependent expression of *rsmB*. Nonetheless, it is obvious that repression mediated by ExpR2 is stronger than that of ExpR1 because the presence of the ExpR2 receptor (observed in an *expI expR1* double mutant) is sufficient to fully repress *rsmB* expression, while ExpR1 is only responsible for a slight reduction in the *rsmB* levels (as observed in the *expI expR2* double mutant). These results differ from those obtained with the *rsmA*::*gfp* promoter fusion, which show that, in accordance with previous work (24), the transcription of *rsmA* is regulated equally by ExpR1 and ExpR2 (see Fig. S3). Because in other bacteria, like *Pseudomonas fluorescens* and *Escherichia coli*, RsmA/CsrA proteins were proposed to influence the expression of their cognate regulatory RNAs through feedback mechanisms (29–31), we investigated whether the AHL-dependent regulation of *rsmB* could take place via RsmA. To test this possibility, we constructed an *rsmA* deletion in the *expI* background. As in other bacteria, the *rsmA* mutant exhibits a severe growth defect (see Fig. S2), making it difficult to compare expression with other mutants (17). To overcome this problem, we selected for a spontaneous mutant in the *expI rsmA* full-deletion background that grows similarly to the WT. This mutant, which we named *rsmA*^sup^ *expI*, is an *rsmA expI* double-deletion mutant with a second-site mutation that partially rescues the growth defect of the *rsmA expI* mutant (see Fig. S2). Our results showed that expression of P_*rsmB*_::*gfp* in the *rsmA*^sup^ *expI* mutant was higher than in the *expI* mutant, indicating that RsmA might repress *rsmB* transcription. However, since RsmA is a posttranscriptional regulator, this regulation is likely to occur indirectly via a transcriptional activator of *rsmB*. Importantly, in the *rsmA*^sup^ *expI* mutant, expression of P_*rsmB*_::*gfp* still responded to AHLs (Fig. 5B), providing evidence that the AHL-dependent regulation of *rsmB* transcription still occurs in the absence of RsmA. Next, we determined if the RsmA-independent control of *rsmB* transcription is dependent on ExpR1 and/or ExpR2. We constructed two additional mutants, the *rsmA*^sup^ *expI expR1* and *rsmA*^sup^ *expI expR2* mutants, and analyzed their P_*rsmB*_::*gfp* expression levels in the presence or absence of AHLs. Our results demonstrated that in the mutant where *expR2* was still present (the *rsmA*^sup^ *expI expR1* mutant), *rsmB* expression still responded to the addition of 3-oxo-C8-HSLs, while in the mutant expressing only ExpR1, addition of 3-oxo-C8-HSL had no effect on *rsmB* levels (Fig. 5). These results, together with the results presented in Fig. S3, show that while in the presence of RsmA, both ExpR1 and ExpR2 can mediate the AHL-dependent regulation of *rsmB*, in the absence of RsmA, ExpR1 no longer regulates *rsmB* expression in response to AHLs, while ExpR2 maintains this regulation. Therefore, we concluded that ExpR2, but not ExpR1, is the regulator responsible for the AHL-dependent regulation of RsmB in the absence of RsmA. We identified one potential ExpR binding site (*lux* box) upstream of *rsmB* (see Fig. S5), which indicates that ExpR2 can regulate *rsmB* transcription directly, but further work is necessary to prove this interaction.

10.1128/mBio.00398-17.2 FIG S2  Growth curves of *P. wasabiae* SCC3193 *expI*, *rsmA expI*, and *rsmA*^sup^ *expI* mutants. The *rsmA expI* mutant has a severe growth defect that is partially restored in the *rsmA*^sup^ *expI* mutant. *n* = 3. Download FIG S2, PDF file, 0.2 MB. Copyright © 2017 Valente et al. 2017 Valente et al. This content is distributed under the terms of the Creative Commons Attribution 4.0 International license.

10.1128/mBio.00398-17.3 FIG S3  Activation of P_*rsmA*::*gfp*_ in WT *P. wasabiae* SCC3193 and *expI*, *expI expR1*, *expI expR2*, and *expI expR1 expR2* mutants. Complementation with AHLs was performed with 2 µM 3-oxo-C8-HSL. Cells were collected when cultures reached an OD_600_ of approximately 3.5. a.u., arbitrary units. Error bars represent the standard deviation of the mean. *n* = 7. Download FIG S3, PDF file, 0.3 MB. Copyright © 2017 Valente et al. 2017 Valente et al. This content is distributed under the terms of the Creative Commons Attribution 4.0 International license.

As discussed below, this level of regulation mediated by ExpR2 and not ExpR1 provides the first evidence that these regulators actually regulate different targets. Former studies reported that ExpR1 and ExpR2 have the same regulatory response to different signal inputs to repress virulence by directly activating the transcription of *rsmA* (11) (see Fig. S3), and there was no previous evidence that these regulators had other targets besides RsmA (11, 32). However, our results show that ExpR2, and not ExpR1, regulates *rsmB* transcription in an RsmA-independent manner.

### AHLs are sufficient to fully restore virulence in a *gacA* mutant.

**FIG 6:**
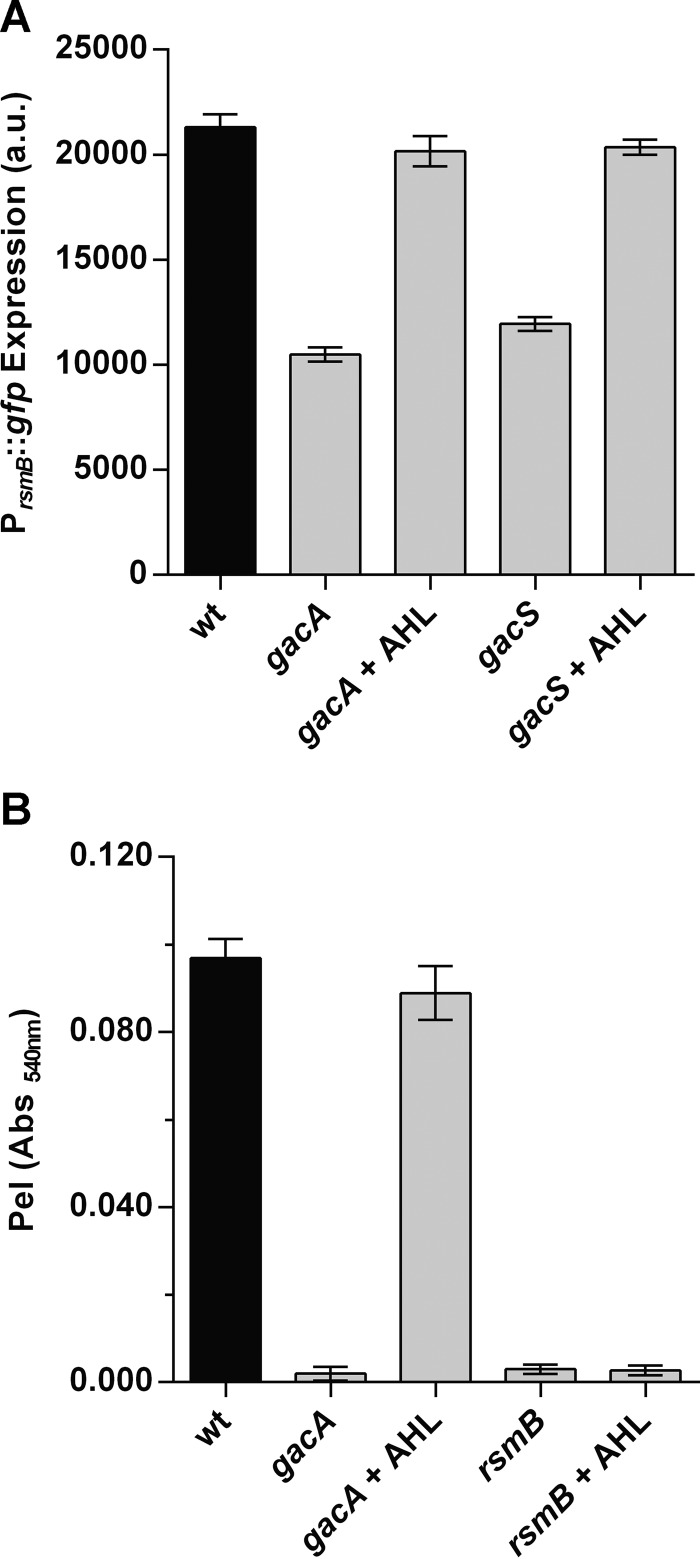
AHL complementation restores *rsmB* expression and Pel activity to WT levels in the absence of the GacS/GacA two-component system. (A) Effects of AHLs on P_*rsmB*_::*gfp* expression in *P. wasabiae* SCC3193 *gacA* and *gacS* mutants compared to that in the WT. (B) Effects of AHLs on Pel activity in *P. wasabiae* SCC3193 *gacA* and *rsmB* mutants compared to that in the WT. Complementation with AHLs was performed with 2 µM 3-oxo-C8-HSL. Cells were collected when cultures reached an OD_600_ of approximately 3.5. a.u., arbitrary units. Error bars represent the standard deviation of the mean. *n* = 7. Abs, absorbance.

Our results showed that AHLs regulate RsmB. This finding, together with the fact that the levels of AHLs are low in *gacS* and *gacA* mutants (see Fig. S1), prompted us to determine if exogenously supplied AHLs could restore *rsmB* transcription and virulence in GacS/GacA system mutants. Addition of 3-oxo-C8-HSL to a *gacA* or *gacS* mutant restored *rsmB* expression to levels similar to those of the WT strain (Fig. 6A). Subsequently, we corroborated that PCWDE activity could also be rescued to WT levels in a *gacA* mutant by the addition of 3-oxo-C8-HSLs (Fig. 6B). However, and in agreement with our results indicating that RsmB plays a major role in this regulation, addition of 3-oxo-C8-HSL to the *rsmB* mutant could not restore Pel activity (Fig. 6B). These results showed that while these two signaling networks of *P. wasabiae* tightly control virulence expression, exogenous addition of AHLs could bypass the requirement for activation of the GacS/GacA system.

### Cross-species activation of RsmB by AHL-producing bacteria triggers early virulence expression in *P. wasabiae.*

**FIG 7:**
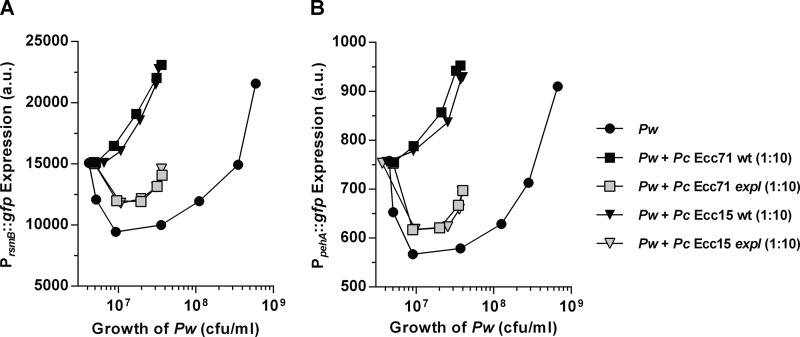
Induction of *rsmB* and *pehA* in multispecies cultures. Shown is the cell density-dependent activation of P_*rsmB*_ (A) and P_*pehA*_ (B) in WT *P. wasabiae* (*Pw*) SCC3193 in the presence of a 10-fold excess of AHL-producing *P. carotovorum* (*Pc*) strain Ecc71 or Ecc15 and the corresponding *expI* (AHL^−^) mutant of *P. carotovorum*. The final densities of *P. wasabiae* are lower in the multispecies cultures than in the monocultures because the yield of *P. wasabiae* is lower in the presence of the other bacteria. This is a representative experiment from three independent experiments. a.u., arbitrary units.

**FIG 8:**
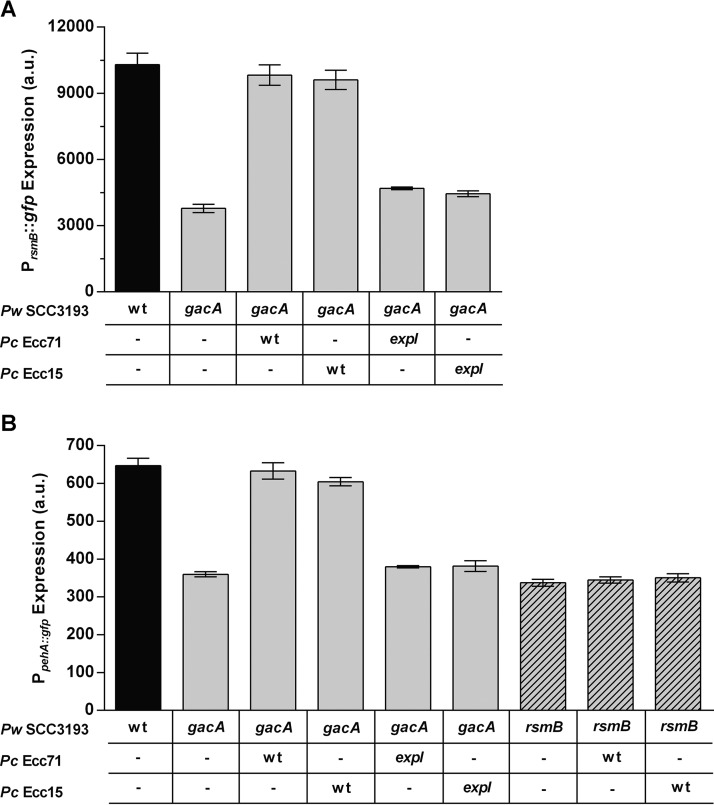
Cross-species induction of *rsmB* and *pehA* in the absence of the Gac system. Activation of P_*rsmB*_ (A) and P_*pehA*_ (B) in *P. wasabiae* SCC3193 *gacA* (gray bars) and *rsmB* (striped bars) mutants in the presence of a 10-fold excess of AHL-producing bacteria and the corresponding *expI* mutants. WT *P. wasabiae* (black bars) and *gacA* and *rsmB* mutants grown in monocultures are shown as references for comparison. *Pw* stands for *P. wasabiae*, and *Pc* stands for *P. carotovorum*. Cells were collected when cultures of the *P. wasabiae* reporter strain reached approximately 5 × 10^7^ CFU/ml. a.u., arbitrary units. Error bars represent the standard deviation of the mean. *n* = 3.

The induction of *rsmB* transcription and virulence in a *gacA* mutant by exogenously supplied AHLs, together with the finding that ExpR2, the receptor capable of sensing foreign AHLs, participates in this regulation, suggested that this level of regulation could be important for cross-species activation in multispecies communities. To test this possibility, we analyzed the expression of *rsmB* and *pehA* with *gfp* promoter fusions in *P. wasabiae* when cocultured with other pectolytic bacteria producing similar AHLs. We compared the density-dependent regulation of *rsmB* and *pehA* expression in a *P. wasabiae* SCC3193 reporter strain in pure culture with that in cocultures with *Pectobacterium carotovorum* Ecc71 and Ecc15, which produce 3-oxo-C6-HSL as the major AHL (12). As shown in Fig. 7, when *P. wasabiae* carrying the P_*rsmB*_::*gfp* or P_*pehA*_::*gfp* plasmid was diluted to a low density in the presence of a 10-fold higher density of *P. carotovorum* Ecc71 or Ecc15, neither the *rsmB* nor the *pehA* gene in *P. wasabiae* was repressed and both continued to be expressed. This response was in contrast to what happened when *P. wasabiae* was growing alone or in the presence of AHL-deficient strain Ecc71 or Ecc15. In cocultures of WT *P. wasabiae* with an *expI* mutant of *P. carotovorum* Ecc71 or Ecc15, a typical density response was observed as the expression of both *rsmB* and *pehA* was turned off at low cell density upon dilution into fresh medium. These results indicate that when grown in coculture with Ecc71 or Ecc15, *P. wasabiae* no longer interprets only its own cell density but instead, by sensing the overall concentration of AHLs present in the environment, induces virulence according to the total density of the related and unrelated AHL-producing bacteria. We next confirmed that this cross-species activation by AHL-producing bacteria that enabled the activation of *rsmB and pehA* transcription in *P. wasabiae* at low cell densities was independent of GacA. As demonstrated above (Fig. 2A and 3), when *P. wasabiae* was grown in pure culture, the expression of both *pehA* and *rsmB* was much lower in the *gacA* mutant than in the WT. However, as shown in Fig. 8, *gacA* was no longer required for induction of *rsmB* or *pehA* when *P. wasabiae* SCC3193 was grown in coculture with either of the two *P. carotovorum* strains tested because induction of *rsmB* and *pehA* was observed when the *P. wasabiae gacA* mutant, carrying the reporter plasmid, was mixed with a 10-fold higher density of *P. carotovorum* Ecc71 or Ecc15 (Fig. 8). These inductions were dependent on the AHLs produced by the two *P. carotovorum* species, as induction of the two *P. wasabiae* reporters was not observed in the cocultures with *expI* mutants of *P. carotovorum* Ecc71 and Ecc15 (Fig. 8). Importantly, *rsmB* was essential for the AHL-dependent response in multispecies cultures, as no activation was observed in the *rsmB* mutant, even in the presence of AHL-producing pectobacteria (Fig. 8B). Furthermore, consistent with the results showing that AHL-dependent regulation of RsmB requires ExpR2 but not ExpR1 (Fig. 5B), induction of *rsmB* by the AHLs produced by *P. carotovorum* Ecc71 was still observed when the *P. wasabiae expR1* mutant was cocultured with *P. carotovorum*, but not when the *P. wasabiae expR2* mutant was used (see Fig. S4).

10.1128/mBio.00398-17.4 FIG S4  Cross-species induction of *rsmB* requires *expR2*. Activation of P_*rsmB*::*gfp*_ in *P. wasabiae* (*Pw*) SCC3193 *expR1* and *expR2* mutants in the presence of WT *P. carotovorum* (*Pc*) Ecc71 and the corresponding *expI* mutant. Cells were collected when cultures of the *P. wasabiae* reporter strain reached approximately 5 × 10^7^ CFU/ml. a.u., arbitrary units. Error bars represent the standard deviation of the mean. *n* = 4. Download FIG S4, PDF file, 0.2 MB. Copyright © 2017 Valente et al. 2017 Valente et al. This content is distributed under the terms of the Creative Commons Attribution 4.0 International license.

These results demonstrated that although the GacS/GacA two-component system was essential for activation of full expression and production of PCWDEs in *P. wasabiae* monocultures, these quorum-sensing-regulated traits could be activated in *P. wasabiae* via RsmB in multispecies cultures, even in the absence of the GacS/GacA system if the other species produced related AHLs. Therefore, these results show that cross-species activation in *P. wasabiae* by other AHL-producing bacteria can override the need for the GacS/GacA system and induce virulence even before the cell density of *P. wasabiae* has reached the quorum threshold.

### *P. wasabiae* virulence is higher in multispecies lesions with AHL-producing bacteria.

**FIG 9:**
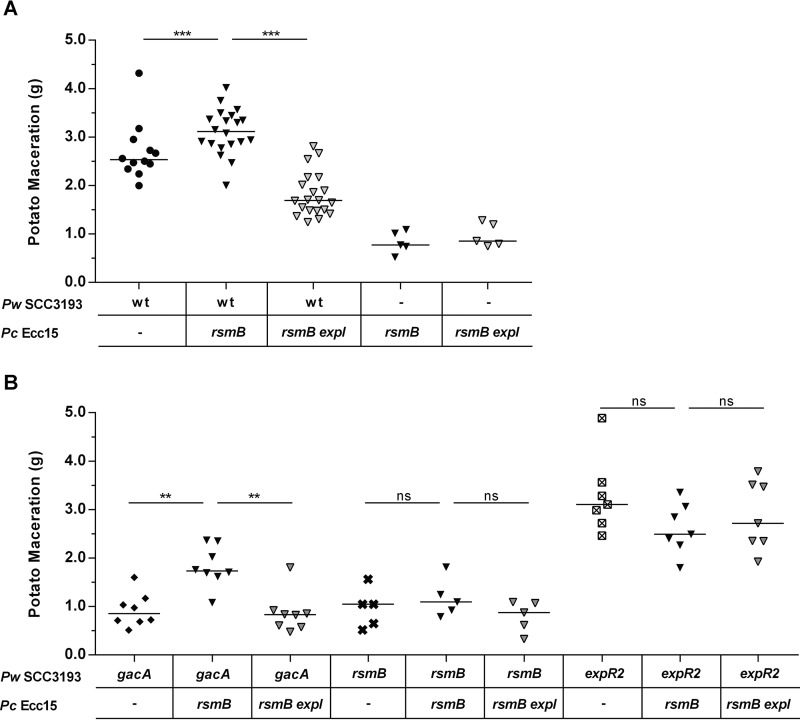
The virulence in *P. wasabiae* is higher in AHL-producing multispecies potato lesions and requires *rsmB*. Comparison of tissue maceration in potato tubers at 24 h caused by WT *P. wasabiae* (*Pw*) SCC3193 (A) and the *gacA*, *rsmB*, and *expR2* mutants (B) in monoinfections or in coinfections with a 10-fold excess of an AHL-producing (Ecc15 *rsmB*) or nonproducing (Ecc15 *rsmB expI*) *P. carotovorum* (*Pc*) strain. The maceration phenotypes of Ecc15 *rsmB* and *rsmB expI* mutants are also shown in panel A. **, *P* ≤ 0.01; ***, *P* < 0.001; ns, not significant (Mann-Whitney test; *n* ≥ 5). Holm-Bonferroni correction was used for multiple comparisons.

We finally asked whether the ability of *P. wasabiae* to respond to foreign AHLs would have an effect on the enzymatic maceration of plant tissue by *P. wasabiae* in potatoes infected with a mixture of *P. wasabiae* and *P. carotovorum* Ecc15 (at a 1:10 ratio). To assess tissue maceration caused by induction of the expression of PCWDEs produced only by *P. wasabiae*, avirulent Ecc15 *rsmB* mutant or *rsmB expI* double mutant bacteria (Fig. 9A) were used in the mixture. It was clear that production of PCWDEs by *P. wasabiae* was higher in tubers infected with mixtures of *P. wasabiae* and *P. carotovorum* Ecc15 capable of producing AHLs (WT *P. wasabiae* plus *P. carotovorum rsmB*) than in the potatoes with mixtures of *P. wasabiae* and the Ecc15 strain that does not produce AHLs (WT *P. wasabiae* plus *P. carotovorum rsmB expI*) (Fig. 9A). Moreover, potatoes infected with *P. wasabiae* monocultures (WT *P. wasabiae*) showed lower levels of maceration than potatoes infected with the same number and density of *P. wasabiae* cells in the mixture with AHL-producing strain Ecc15 (WT *P. wasabiae* plus *P. carotovorum rsmB*) (Fig. 9A). We further confirmed that, in line with our *in vitro* data (Fig. 8B), the AHL-mediated activation of *P. wasabiae* virulence observed in the multispecies infections with the Ecc15 strain that produces AHLs is independent of GacA, as the virulence of a *P. wasabiae gacA* mutant could still be induced by the AHLs produced by Ecc15 (Fig. 9B). Importantly, both *rsmB* and *expR2* were essential for the AHL-dependent response in multispecies infections, as no additional induction of virulence was observed in the *P. wasabiae rsmB* or *expR2* mutant either in the absence or in the presence of AHL-producing Ecc15 bacteria (Fig. 9B).

## DISCUSSION

In this study, we analyzed the integration of the two major signaling networks controlling virulence in *P. wasabiae*. Our results showed that the AHL synthase ExpI, as well as all of the components of the Gac/Rsm system, is essential for activation of virulence in this bacterium. These findings provided evidence of a previously unreported link between these two signaling networks. Indeed, our studies revealed that the Gac/Rsm pathway affects the levels of AHLs, but most importantly, we discovered that AHLs control the levels of the regulatory RNA RsmB. This additional level of regulation provides an explanation for the avirulent phenotype of *expI*. In the absence of AHLs, ExpR2 represses *rsmB* and activation of the GacS/GacA system is not sufficient to relieve this inhibition. These results also showed that ExpR1 and ExpR2 regulate different targets. Earlier studies identified RsmA as the only known target of ExpR1 and ExpR2. We show that while ExpR1 and ExpR2 regulate RsmA expression to similar levels, only ExpR2 is required for AHL-dependent regulation of *rsmB* transcription. Given the positions of *expR1* and *expR2* in the genome, where *expR1* is linked to *expI* and *expR2* is located elsewhere, and the fact that while ExpR1 is specific to 3-oxo-C8-HSL, ExpR2 also responds to AHLs produced by other species, it is thought that ExpR1 is the original partner of ExpI and that a later acquisition of ExpR2 probably enabled the gain of a novel function (11). On the basis of our results, we speculate that the RsmA-independent control of ExpR2 on *rsmB* expression could be the novel function, or at least one of the novel functions, that provided a potential benefit of maintaining ExpR2.

The discovery of the regulation of *rsmB* expression through AHLs led us to show that exogenously supplied AHLs could override the requirement of the GacS/GacA system inducing *rsmB* expression. This regulation of *rsmB* in *P. wasabiae* is crucial for the response to AHLs in this bacterium because virulence can be restored to WT levels by the addition of AHLs in all of the mutants tested, except in the *rsmB* mutant. Overall, these results demonstrate that RsmB, previously thought to be regulated mainly by the GacS/GacA two-component system, is a key component in the integration of the sensory information contained in the AHL and GacS/GacA signaling pathways in *P. wasabiae*.

The finding that ExpR2, the receptor that enables *P. wasabiae* to respond to AHLs from other species, has an additional function in the AHL-dependent regulation of RsmB that differentiates it from ExpR1 led us to hypothesize that having RsmB under the control of the AHL system could be relevant in multispecies communities. The further evidence that addition of AHLs was sufficient to restore the induction of Pel activity in a *gacA* mutant prompted us to test whether cross-species activation by AHL-producing bacteria could override the need for the GacS/GacA system in *P. wasabiae*. We examined *rsmB* and *pehA* expression in various *P. wasabiae* reporter strains mixed with a higher cell density of two *P. carotovorum* strains (Ecc71 and Ecc15). Unlike *P. wasabiae*, which produces mainly 3-oxo-C8-HSL as the main AHL, *P. carotovorum* produces 3-oxo-C6-HSL as the major AHL (12). We observed that although *P. wasabiae gacA* and *rsmB* mutants had low levels of PCWDEs in monocultures, in multispecies cultures with WT *P. carotovorum*, *pehA* expression was induced in the *P. wasabiae gacA* mutant by the AHLs produced by *P. carotovorum* but not in the *P. wasabiae rsmB* mutant. Therefore, RsmB, but not GacA, was essential for the AHL-dependent cross-species activation of virulence. These findings indicate that by overriding the need to activate the GacS/GacA system in multispecies cultures, WT *P. wasabiae* is primed, independently of its own population density, to induce virulence expression even at very low cell densities in response to the AHLs produced by other species. On the contrary, under conditions where no other AHL-producing bacteria are present, the GacS/GacA two-component system, which also regulates the rate of AHL production, might be important to provide the necessary buffer to delay activation of the production of virulence factors (such as PCWDEs), ensuring that the population engages in the production of these common goods only when the quorum has been reached and thus when it is productive to do so.

We speculate that integration of these two signal transduction networks in *P. wasabiae* could have evolved to provide a fast response to changing ecological situations. Different pathogen species are often isolated from diseased tubers within the same affected crops (33) and the same potato tubers (34). Therefore, it is plausible that multispecies lesions can occur in nature. Under such a hypothetical scenario, it is conceivable that, in soil, *P. wasabiae* will encounter plants already colonized by large numbers of related AHL-producing bacteria. In such niches, *P. wasabiae*, and probably other pectobacteria, might profit by responding to AHLs produced by others and by being primed to join the community in the effort to degrade the plant cell wall barrier. It is not obvious whether cooperation or competition is more likely to take place among the different bacteria within lesions, but sensing the presence of other species might be beneficial in both scenarios. These arguments are supported by our observation that *P. wasabiae* can induce the expression of virulence by sensing AHLs produced by other bacteria present at higher concentrations in multispecies potato lesions and by the fact that potato maceration caused by induction of virulence in *P. wasabiae* was higher in the multispecies lesions than in the lesions infected with the same cell densities of *P. wasabiae* in monocultures. It has been suggested that transmission of soft rot disease is driven by insect vectors harboring important plant pathogens (35, 36), with these insects being easily attracted to decaying plant material. Therefore, bigger lesions should increase the chances of attracting insects and other organisms that can serve as transmission vectors. Moreover, transmission could be facilitated by direct contamination through spreading of rotting material to adjoining tubers. Therefore, we reason that higher tissue maceration is potentially better for bacterial transmission and thus bacterial fitness.

Interestingly, an extra layer of regulation ensures that the signaling pathways regulating virulence in *P. wasabiae* respond to quorum-sensing signals only in the presence of damaged plant tissue. This level of virulence regulation is provided by the regulator KdgR, which represses the transcription of PCWDEs in the absence of plant signals (30, 37). The presence of plant cell wall breakdown products (supplied in our *in vitro* experiments by the addition of polygalacturonic acid [PGA] to the culture medium) is required to relieve KdgR repression and activate virulence. In *P. wasabiae*, this level of regulation might be essential also to ensure that the population will not respond to all AHL-producing bacteria. Because of the KdgR control, by responding to AHLs only in the presence of plant signals, presumably, *P. wasabiae* will only engage with microorganisms that are also participating in the degradation of the plant tissue. The multiple levels of regulation to control virulence described here show how signal transduction networks are tightly regulated to ensure that virulence is only activated in the right context and illustrate how ecological interactions can have such an important role in the regulation of virulence.

*Vibrio cholerae* and *Vibrio harveyi* are other examples of bacteria where different cell-to-cell signals are integrated into a single-output pathway (38, 39). In a recent report, Jung et al. demonstrated that in *V. cholerae*, the absence of any of the four signal receptors can be compensated for by the other three, maintaining *in vivo* colonization levels similar to those of the WT (38). Thus, in contrast to what happens in *P. wasabiae* (Fig. 1), in *V. cholerae*, one signaling system is enough to induce WT colonization levels in the infection assays used (38). Moreover, another difference stems from the fact that WT *V. cholerae* appears highly resistant to signal perturbation, as an exogenous supply of the cholera autoinducer could not induce a response as long as the other systems were present. These findings were interpreted as a strategy adopted by *V. cholerae* to be less susceptible to environmental interference, in particular, to signals from other bacteria (38). We concluded that in pectobacteria, the architecture of the cell-to-cell communication networks functions contrary to that of *V. cholerae*, as it allows input from species in the community to alter gene expression.

The present study and studies of *Vibrio* spp. exemplify how bacteria use different cell-to-cell signaling architectures to provide alternative outputs, presumably to optimize survival in their different ecological niches (40–42). We argue that our work supports the conclusion that the ecological factors are likely to be among the major factors dictating the constraints on the architecture integrating multiple quorum-sensing signal networks. Bacteria may have evolved to respond to the presence of other species, as this might be the case in *P. wasabiae*, which responds to other pectolytic bacteria, or to have robust systems that are optimized to avoid interference from neighbors, as has been proposed for *V. cholerae*. Therefore, our expectations are that analyses of the signaling networks such as the one described here will help us to understand how different species react to their neighbors and respond under different ecological conditions.

## MATERIALS AND METHODS

### Bacterial strains, plasmids, and culture conditions.

The strains, plasmids, and primers used in this study are listed in Tables S1 and S2. All of the *P. wasabiae* strains used are derived from SCC3193 (28). *P. wasabiae* and *P. carotovorum* strains were grown at 30°C with aeration in Luria-Bertani (LB) broth supplemented with 0.4% PGA (Sigma P3850). PGA is required to induce the expression of virulence. *E. coli* DH5α was used for routine cloning procedures. This strain was grown at 37°C with aeration in LB broth. Antibiotics were used at the following concentrations (mg liter^−1^): ampicillin, 100; kanamycin, 50; spectinomycin (Spec), 50; chloramphenicol, 25; streptomycin, 100.

10.1128/mBio.00398-17.7 TABLE S1  Strains and plasmids used in this study. Download TABLE S1, PDF file, 0.6 MB. Copyright © 2017 Valente et al. 2017 Valente et al. This content is distributed under the terms of the Creative Commons Attribution 4.0 International license.

10.1128/mBio.00398-17.8 TABLE S2  Primers used in this study. Download TABLE S2, PDF file, 0.4 MB. Copyright © 2017 Valente et al. 2017 Valente et al. This content is distributed under the terms of the Creative Commons Attribution 4.0 International license.

### Genetic and molecular techniques.

To construct the P_*rsmB*_::*gfp* reporter (see Fig. S5), the fragment containing the P_*rsmB*_::*gfp* fusion was amplified from pRSV206 (21) with P0528 and P0665 and cloned into pOM1 (43). To construct the P_*rsmA*_::*gfp* reporter, the promoter region of *rsmA* was amplified with primers P0766 and P0656 and cloned into pUC18 (44). The *gfp* fragment obtained by PCR from pCMW1 (45) was cloned downstream of pUC18-P_*rsmA*_. Subsequently, the P_*rsmA*_::*gfp* fragment was amplified with P0766 and P0665 and cloned into pOM1. To construct the P_*pehA*_::*gfp* reporter, the promoter region of *pehA* from SCC3193 was amplified with P1028 and P0658 and cloned into pUC18. Subsequently, the *gfp* fragment obtained by PCR from pCMW1 was cloned downstream of pUC18-P_*pehA*_. Next, the P_*pehA*_::*gfp* fragment was amplified with P1028 and P0665 and cloned into pOM1. *P. wasabiae* SCC3193 deletion mutants were constructed by chromosomal gene replacement with an antibiotic marker with the λ-Red recombinase system (46, 47) as previously described (21). For *rsmB*::*kan*, *expR1*::*kan*, and *expR2*::*kan* deletions, plasmid pKD4 (47) was used as the template to amplify the *kan* gene. The *rsmA* suppressor (*rsmA*^sup^ *expI*; see Table S3) was obtained by selection of overgrown spontaneous mutants in LB agar plates and used as recipients for electroporation of the *expR1*::*kan* or *expR2*::*kan* fragment. Similarly to *P. wasabiae* SCC3193, *P. carotovorum* Ecc15 deletion mutants were also constructed by chromosomal gene replacement with an antibiotic marker. The plasmid used to express the arabinose-inducible λ-Red recombinase system (pLIPS) in Ecc15 was constructed by introducing the system into the pOM1 vector. The λ-Red recombinase system (composed of the *araC*, *gam*, *bet*, *exo*, and *af60A* genes, a total of 3,421 bp) was amplified by PCR from vector pKD46 with primers P1108 and P1109 and cloned into pOM1.

10.1128/mBio.00398-17.5 FIG S5  Sequence of the promoter fusion P_*rsmB*_::*gfp*. The nucleotide sequence of the promoter fusion used to quantify the expression of RsmB is shown. In black is the nucleotide region of the *rsmB* promoter and flanking regions cloned into the pCMW1. Underlined are the sequences corresponding to a potential ExpR binding site (*lux* box) identified by Softberry software (bprom), the predicted −35 and −10 sequences and +1 site based on homology with *P. carotovorum* (2), and a consensus RsmA binding site (3). The plasmid sequence is gray. The ribosomal binding site (RBS) included in the plasmid and the GFP sequence are also gray but underlined. See Text S1 for references. Download FIG S5, PDF file, 0.8 MB. Copyright © 2017 Valente et al. 2017 Valente et al. This content is distributed under the terms of the Creative Commons Attribution 4.0 International license.

10.1128/mBio.00398-17.9 TABLE S3  Sequence of the *rsmA*^sup^ *expI* mutant. Download TABLE S3, PDF file, 0.5 MB. Copyright © 2017 Valente et al. 2017 Valente et al. This content is distributed under the terms of the Creative Commons Attribution 4.0 International license.

PCRs for cloning purposes were performed with the proofreading Herculase II polymerase (Agilent) for SCC3193 or with Bio-X-ACT (Bioline) for Ecc15. Other PCRs were performed with Dream *Taq* polymerase (Fermentas). Digestions were done with Fast Digest Enzymes (Fermentas), and ligations were performed with T4 DNA ligase (New England Biolabs). All cloning steps were performed with *E. coli* DH5α. All mutants and constructs were confirmed by sequencing at the Instituto Gulbenkian de Ciência sequencing facility.

### Pectate lyase activity assay.

To analyze extracellular pectate lyase (Pel) activity, overnight cultures were diluted to a starting OD_600_ of 0.05 in LB broth supplemented with 0.4% PGA. At the time points indicated, aliquots were collected to evaluate growth and to analyze Pel activity in the supernatants by the previously described thiobarbituric acid colorimetric method (21, 48).

### *P. wasabiae* virulence assay.

Virulence was analyzed by assessing the maceration of potato tubers with the protocol adapted from reference 49. Potatoes were washed and surface sterilized by being soaked for 10 min in 10% bleach, followed by 10 min in 70% ethanol. Overnight cultures in LB broth were washed twice and diluted to an OD_600_ of 0.05 in phosphate-buffered saline (PBS). For multispecies infections, overnight cultures of *P. wasabiae* and *P. carotovorum* were washed twice and diluted in PBS to OD_600_s of 0.05 and 0.5, respectively, separately or in mixtures. Thirty-microliter aliquots were then used to inoculate the previously punctured potatoes. Potato tubers were incubated at 28°C at a relative humidity above 90% for 24 or 48 h, as specified. After incubation, potatoes were sliced and macerated tissue was collected and weighed.

### Promoter expression analyses.

*P. wasabiae* strains containing the different promoter-*gfp* plasmids were grown overnight in LB broth plus PGA and Spec and inoculated into fresh medium at a starting OD_600_ of 0.05. At the times indicated, aliquots were collected to monitor growth and assess gene expression. As previously described (21), for the analyses of gene expression, aliquots of the cultures were diluted 1:100 in PBS and expression was measured by flow cytometry (LSRFortessa; BD) and analyzed with Flowing Software v 2.5.1. A minimum of 5,000 green fluorescent protein (GFP)-positive single cells were acquired per sample. Expression of the promoter-*gfp* fusions is reported as the median GFP expression of GFP-positive single cells in arbitrary units.

### Cross-species expression experiments.

The interspecies signaling effects on the activation of *rsmB* expression were tested similarly to those of single species. Briefly, WT and *gacA* and *rsmB* mutant *P. wasabiae* strains harboring either plasmid *P*_*rsmB*_::*gfp* or P_*pehA*_::*gfp* and WT and *expI* mutant *P. carotovorum* Ecc15 and Ecc71 strains were grown overnight in LB broth plus 0.4% PGA. *P. wasabiae* strains (reporters) and *P. carotovorum* strains were diluted in fresh medium to OD_600_s of 0.05 and 0.5, respectively (1:10). The diluted cultures were maintained separately for 2 h to allow the reporter strain to reduce the AHL concentration and mimic the low cell density state. After 2 h, the *P. wasabiae* cells were centrifuged and resuspended in the *P. carotovorum* high cell density cultures. Aliquots of the multispecies cultures were diluted 1:100 in PBS, and expression was measured by flow cytometry as explained above. The two populations were clearly distinguishable by the presence or absence of GFP fluorescence. *P. wasabiae* growth was quantified by determining the number of CFU per milliliter of LB agar supplemented with Spec.

### Statistical analysis.

Data were analyzed with GraphPad Prism 6 software and R program v 3.0.2. The Mann-Whitney test was performed to determine statistical significance, and *P* values were adjusted with the Holm-Bonferroni correction for multiple comparisons. An adjusted *P* value of <0.05 was used as the cutoff for statistical significance.
