# Supplementary figures and images for: Signal Integration in Quorum Sensing Enables Cross-Species Induction of Virulence in Pectobacterium wasabiae
Source: mBio. 2017 May 23;8(3):e00398-17. doi: 10.1128/mBio.00398-17 (PMC5442451; doi:10.1128/mBio.00398-17)

**A**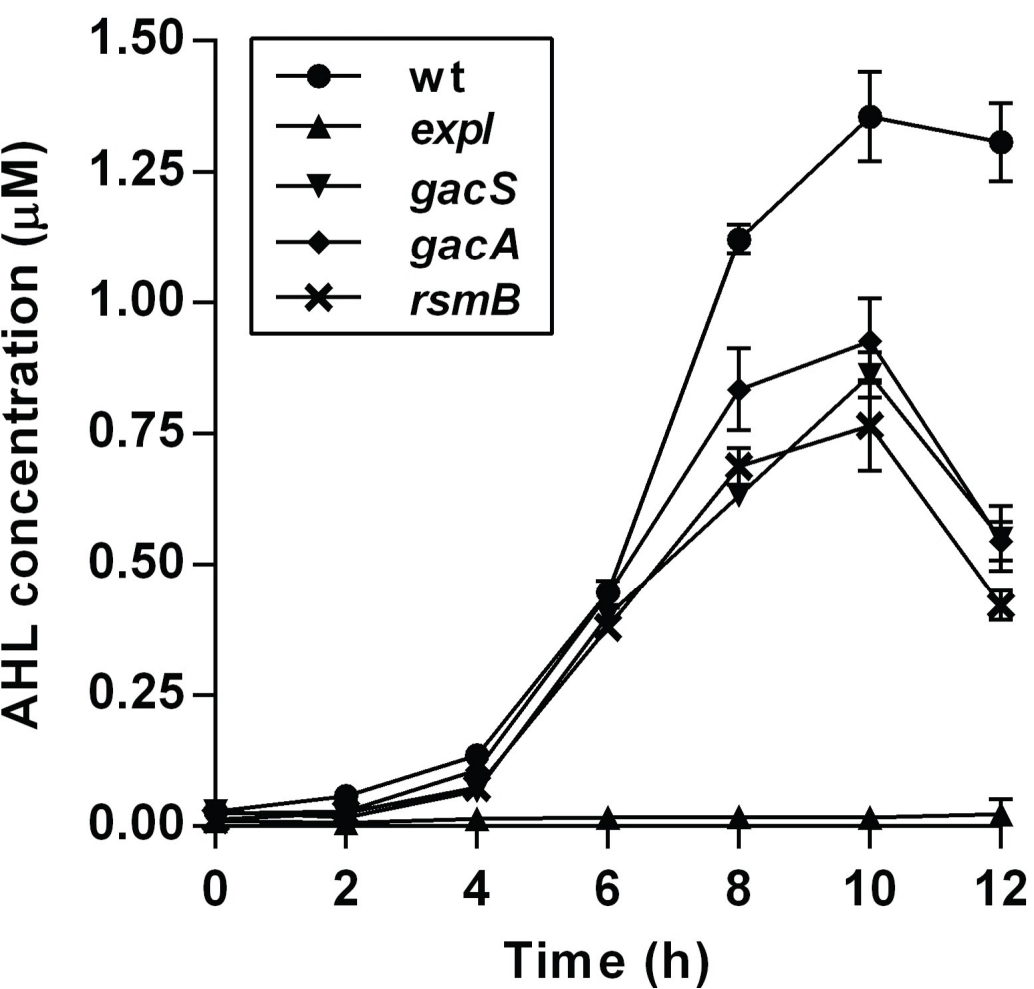**B**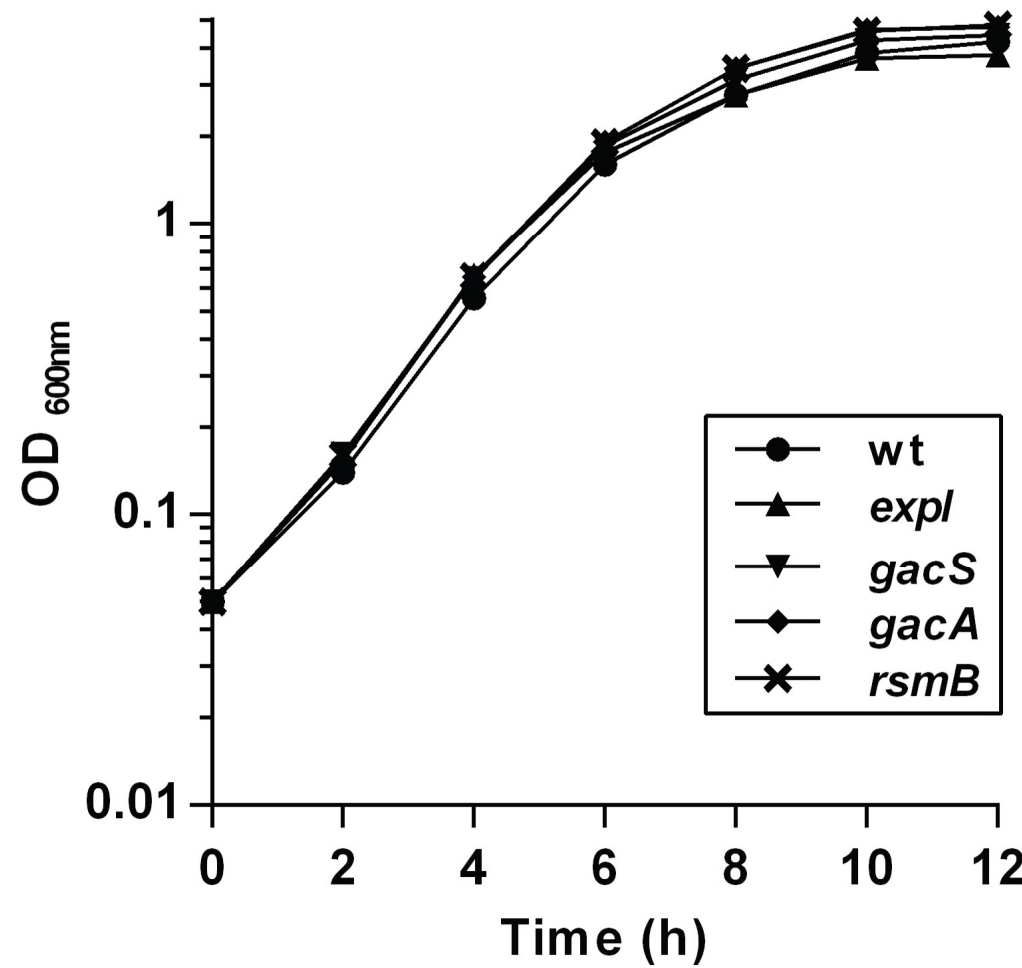

Supplement: FIG S1 [file mbo003173315sf1.pdf]

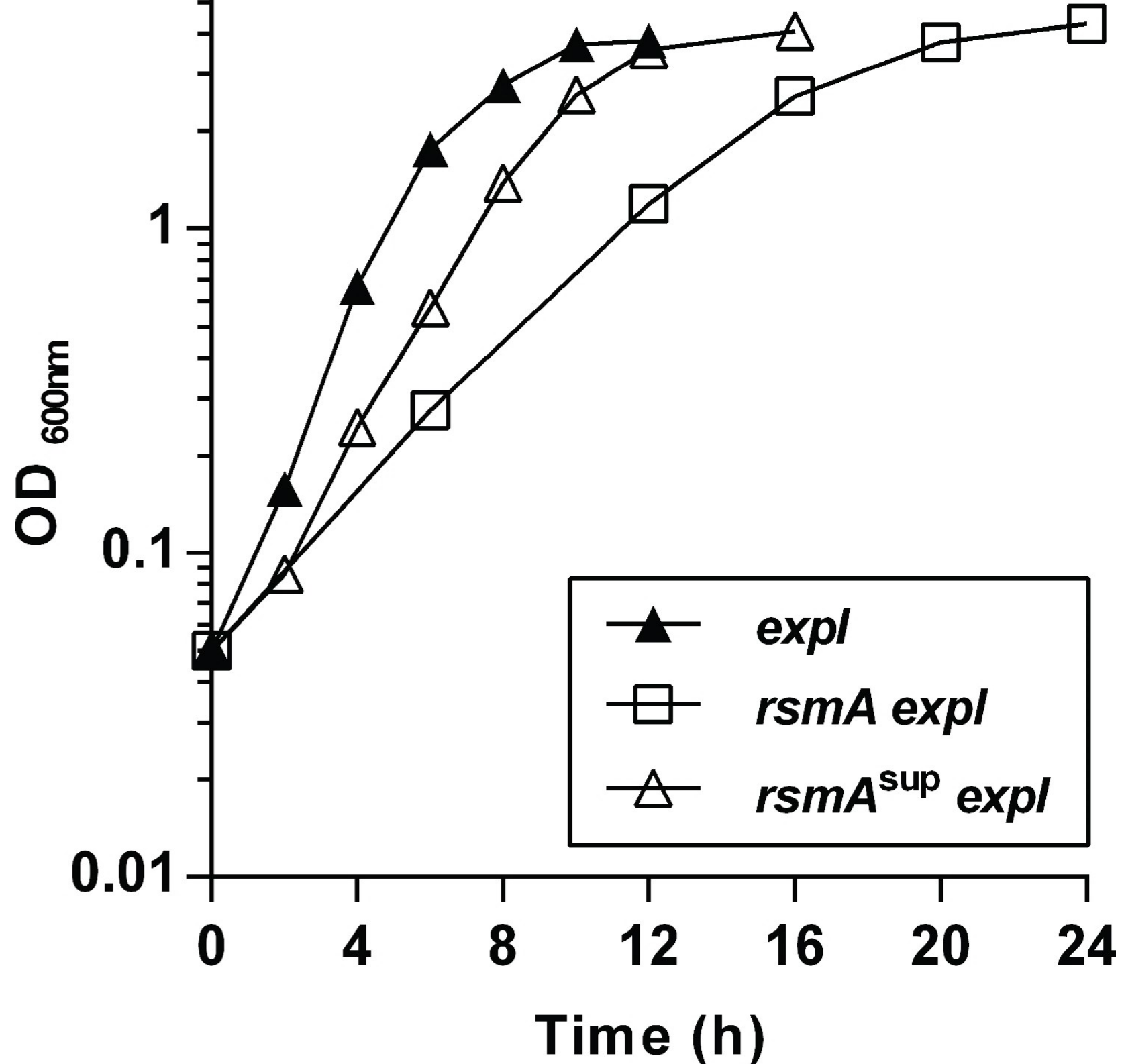

Supplement: FIG S2 [file mbo003173315sf2.pdf]

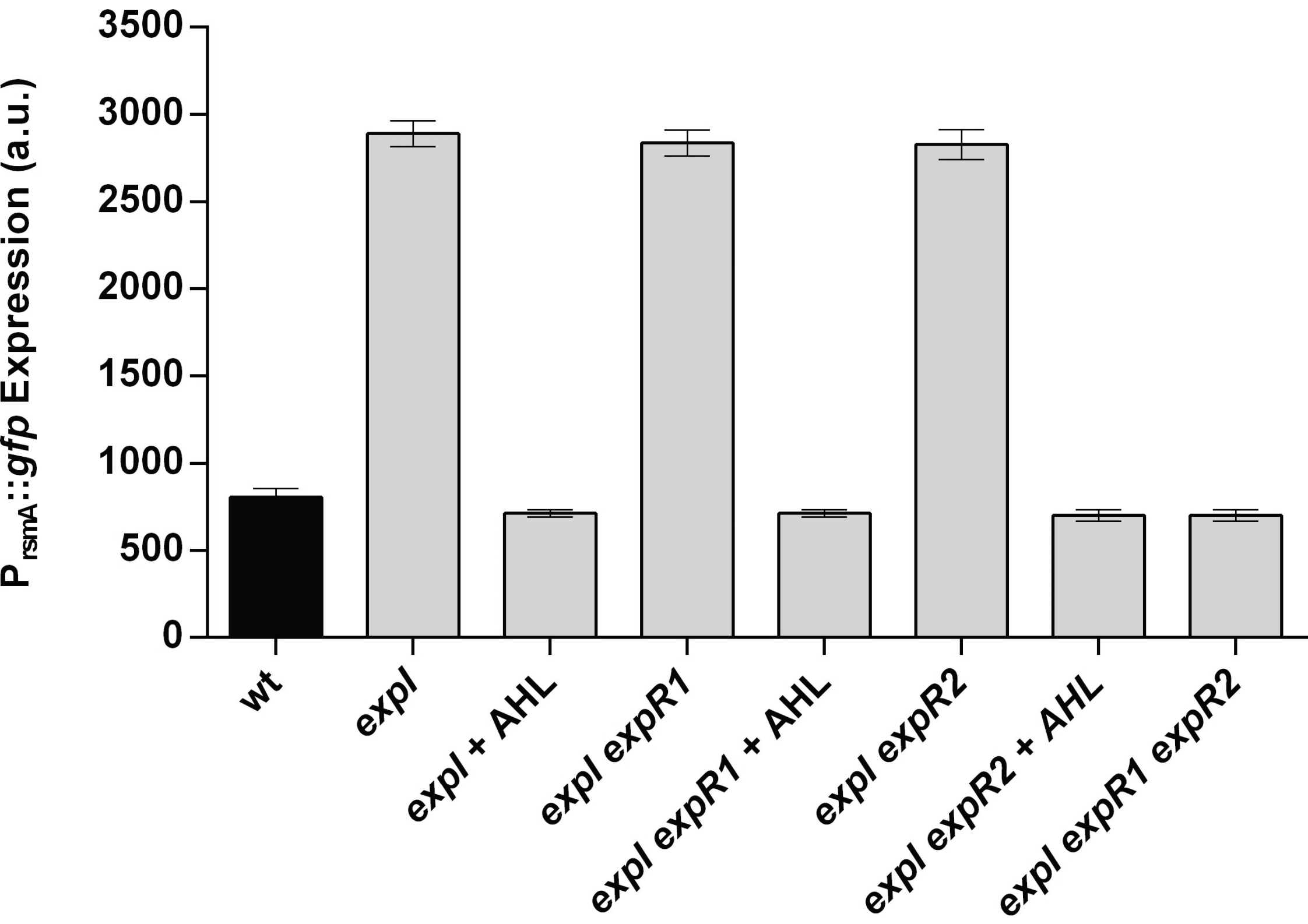

Supplement: FIG S3 [file mbo003173315sf3.pdf]

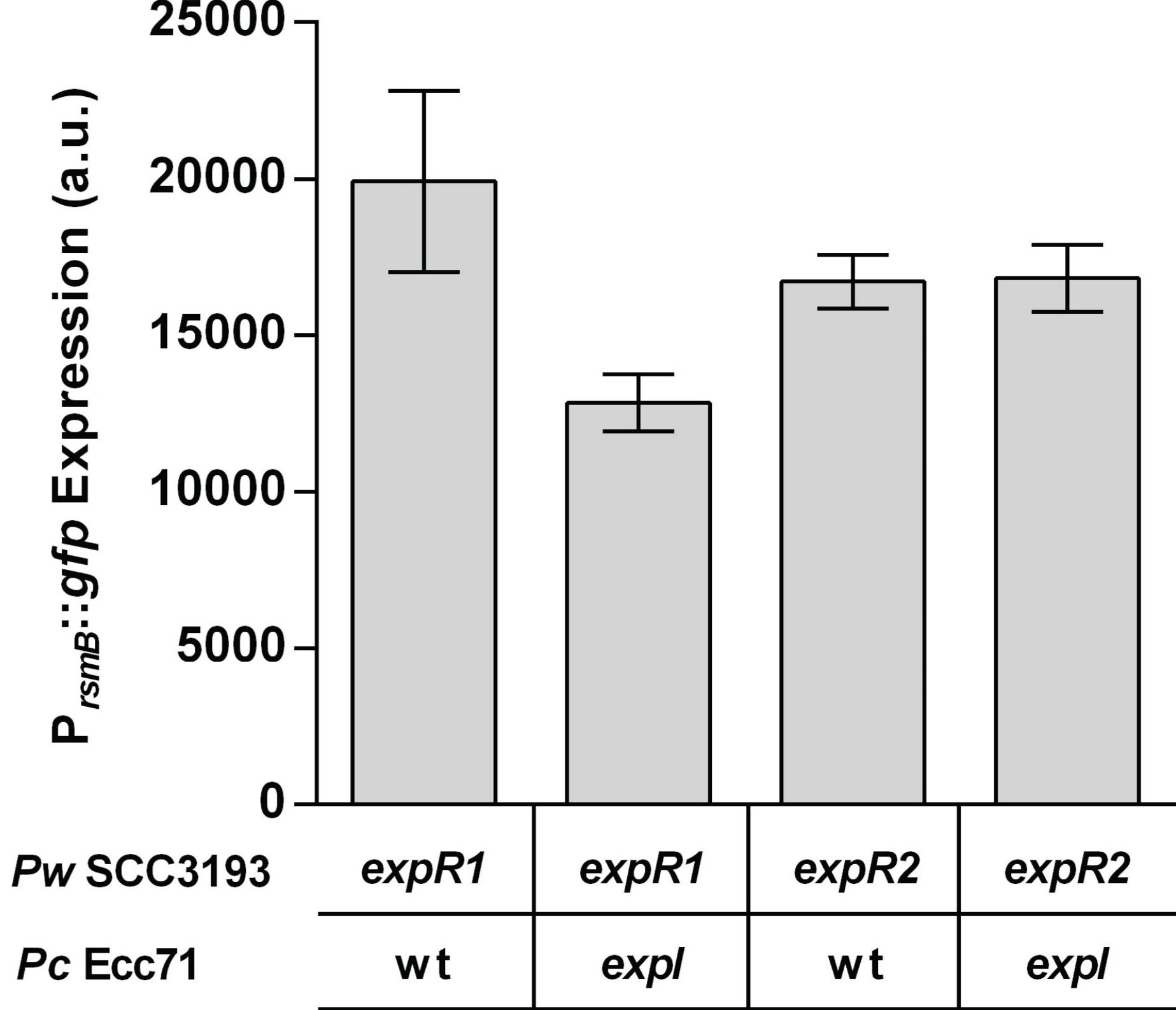

Supplement: FIG S4 [file mbo003173315sf4.pdf]
