# Supplementary material for: Signal Integration in Quorum Sensing Enables Cross-Species Induction of Virulence in Pectobacterium wasabiae
Source: mBio. 2017 May 23;8(3):e00398-17. doi: 10.1128/mBio.00398-17 (PMC5442451; doi:10.1128/mBio.00398-17)
Supplement: TEXT S1 [file mbo003173315s1.pdf]

Supplemental References:

1. Winson MK, Swift S, Fish L, Throup JP, Jørgensen F, Chhabra SR, Bycroft BW, Williams P, Stewart GSAB. 1998. Construction and analysis of luxCDABE-based plasmid sensors for investigating N-acyl homoserine lactone-mediated quorum sensing. FEMS Microbiol Lett 163:185–192.

2. Liu Y, Cui Y, Mukherjee A, Chatterjee AK. 1998. Characterization of a novel RNA regulator of *Erwinia carotovora* ssp. *carotovora* that controls production of extracellular enzymes and secondary metabolites. Mol Microbiol 29:219–234.

3. Kulkarni PR, Jia T, Kuehne SA, Kerkering TM, Morris ER, Searle MS, Heeb S, Rao J, Kulkarni R V. 2014. A sequence-based approach for prediction of CsrA/RsmA targets in bacteria with experimental validation in *Pseudomonas aeruginosa*. Nucleic Acids Res 42:6811–6825.

4. Pirhonen M, Heino P, Helander I, Harju P, Palva ET. 1988. Bacteriophage T4 resistant mutants of the plant pathogen *Erwinia carotovora*. Microb Pathog 4:359–367.

5. Valente RS, Xavier KB. 2016. The Trk potassium transporter is required for RsmB-mediated activation of virulence in the phytopathogen *Pectobacterium wasabiae*. J Bacteriol 198:248–255.

6. Sjöblom S, Brader G, Koch G, Palva ET. 2006. Cooperation of two distinct ExpR regulators controls quorum sensing specificity and virulence in the plant pathogen *Erwinia carotovora*. Mol Microbiol 60:1474–1489.

7. Basset a, Khush RS, Braun A, Gardan L, Boccard F, Hoffmann J a, Lemaitre B. 2000. The phytopathogenic bacteria *Erwinia carotovora* infects *Drosophila* and activates an immune response. Proc Natl Acad Sci U S A 97:3376–3381.

8. Murata H. 1991. Molecular Cloning of an *aepA* Gene that Activates Production of Extracellular Pectolytic, Cellulolytic, and Proteolytic Enzymes in *Erwinia carotovora* subsp. *carotovora*. Mol Plant-Microbe Interact 4:239.

9. Murata H, Chatterjee A, Liu Y, Chatterjee AK. 1994. Regulation of the production of extracellular pectinase, cellulase, and protease in the soft rot bacterium *Erwinia carotovora* subsp. *carotovora*: evidence that *aepH* of *E. carotovora* subsp. *carotovora* 71 activates gene exp. Appl Environ Microbiol 60:3150–3159.

10. Waters CM, Bassler BL. 2006. The *Vibrio harveyi* quorum-sensing system uses shared regulatory components to discriminate between multiple autoinducers. Genes Dev 20:2754–2767.

11. Yanisch-Perron C, Vieira J, Messing J. 1985. Improved M13 phage cloning vectors and host strains: nucleotide sequences of the M13mp18 and pUC19 vectors. Gene 33:103–119.

12. Datsenko KA, Wanner BL. 2000. One-step inactivation of chromosomal genes in *Escherichia coli* K-12 using PCR products. Proc Natl Acad Sci U S A 97:6640–6645.

13. Kaniga K, Delor I, Cornelis GR. 1991. A wide-host-range suicide vector for improving reverse genetics in gram-negative bacteria: inactivation of the *blaA* gene of *Yersinia enterocolitica*. Gene 109:137–141.

14. Espéli O, Moulin L, Boccard F. 2001. Transcription attenuation associated with bacterial repetitive extragenic BIME elements. J Mol Biol 314:375–386.

15. Deatherage DE, Barrick JE. 2014. Identification of Mutations in Laboratory-Evolved Microbes from Next-Generation Sequencing Data Using breseq. Methods Mol Biol 1151:165-188

16. Robinson JT, Thorvaldsdóttir H, Winckler W, Guttman M, Lander ES, Getz G, Mesirov JP. 2011. Integrative genomics viewer. Nat Biotechnol 29:24–26.

17. Li H, Durbin R. 2010. Fast and accurate long-read alignment with Burrows-Wheeler transform. Bioinformatics 26:589–595.
