# Supplementary material for: Signal Integration in Quorum Sensing Enables Cross-Species Induction of Virulence in Pectobacterium wasabiae
Source: mBio. 2017 May 23;8(3):e00398-17. doi: 10.1128/mBio.00398-17 (PMC5442451; doi:10.1128/mBio.00398-17)
Supplement: TABLE S1 [file mbo003173315st1.pdf]

Table S1. Strains and plasmids used in this study.

Strains and Plasmids	Relevant Genotype	Source	Strain name
P. wasabiae			
SCC3193	Wild type	(4)	SCC3193
expI	*expI::cm*	Andreas Mäe (Lab collection)	expI
gacS	*ΔgacS::strep*	This study	RSV471
gacA	*ΔgacA::strep*	(5)	RSV470
rsmB	*ΔrsmB::kan*	This study	RSV091
expR1 expR2	*expR1::cm, expR2::kan*	(6)	SCC907
expI expR1 expR2	*expIexpR1::cm, expR2::kan*	(6)	SCC906
expI rsmB	*expI::cm, ΔrsmB::kan*	This study	RSV528
rsmA	*ΔrsmA::strep*	This study	RSV497
rsmA expI	*ΔrsmA::strep, expI::cm*	This study	RSV531
rsmA^{sup} *expI*	*ΔrsmA::strep* supressor, *expI::cm*	This study	RSV693
rsmA^{sup} *expI expR1*	*ΔrsmA::strep* supressor, *expI::cm, expR1::kan*	This study	RSV769
rsmA^{sup} *expI expR2*	*ΔrsmA::strep* supressor, *expI::cm, expR2::kan*	This study	RSV770
expR1	*expR1::cm*	(6)	SCC5003
expR2	*expR2::kan*	(6)	SCC905
P. carotovorum			
ECC15	Wild type	(7)	Ecc15
	expI	Lab collection	
rsmB	*ΔrsmB::kan*	Lab collection	FDV141
rsmB expI	*ΔrsmB::kan , ΔexpI::cm*	Lab collection	FDV145
ECC71	Wild type	(8)	AC5006
	expI	(9)	AC5090
E. coli			
JM109	JM109 carrying pSB401 (*luxRI'*::*luxCDABE*)	(1)	RSV090
DH5α	Cloning strain	Lab collection	DH5α
**Plasmids**			
pCMW1	Promoterless-*gfp* reporter vector, kan^{r}	(10)	
pRSV206	P_{rsmB}::*gfp* fusion containing the RBS from pCMW1	(5)	
pUC18	Cloning vector, amp^{r}	(11)	

pLIPS	λ Red recombinase expressing vector for Ecc15, spec^r	This study	
pKD46	λ Red recombinase expressing vector, amp^r	(12)	
pKD4	Vector containing kanamycin cassette, kan^r	(12)	
pKNG101	Vector containing streptomycin cassette, strep^r	(13)	
pOM1	Cloning vector, spec^r	(14)	
pOM1-P_{pehA}::*gfp*	Reporter plasmid pOM1 with a P_{pehA}::*gfp* fusion	This study	
pOM1-P_{rsmB}::*gfp*	Reporter plasmid pOM1 with a P_{rsmB}::*gfp* fusion containing the RBS from pCMW1	This study	
pOM1-P_{rsmA}::*gfp*	Reporter plasmid pOM1 with a P_{rsmA}::*gfp* fusion	This study	
pFED347-P_{lac}*rsmB*	Vector expressing *rsmB* under control of the lac promoter	This study	
