# Supplementary material for: Signal Integration in Quorum Sensing Enables Cross-Species Induction of Virulence in Pectobacterium wasabiae
Source: mBio. 2017 May 23;8(3):e00398-17. doi: 10.1128/mBio.00398-17 (PMC5442451; doi:10.1128/mBio.00398-17)
Supplement: TABLE S2 [file mbo003173315st2.pdf]

Table S2. Primers used in this study

**Primer name**	**Description**	**Sequence**
P0528	P*rsmB* amplification (SphI)	GA**GCATGC**TGTCCTGGAAGAATTCGGCACG
P0576	*gfp* amplification (SphI)	CCTTAC**GCATGC**ATGGCTAGCAAAGGAGAAGAACTCT
P0656	P*rsmA* amplification (SphI)	CCTTAC**GCATGC**TCTTTGCTCCTTGAAAGATTATAAAG
P0658	P*pehA* amplification (SphI)	CCTTAC**GCATGC**GTCAAACCTCACCTTATAAATGTC
P0665	*gfp* amplification (BamHI)	CCTTAC**GGATCC**TCAGTTGTACAGTTCATCCATGCCA
P0764	*expR1* amplification (XbaI)	CCTTAC**TCTAGA**TCGCTGCAACTACCGCGCGGCCTTTT
P0765	*expR2* amplification (XbaI)	CCTTAC**TCTAGA**CCGCGAACTCAGTTCCTGCTGCACAT
P0766	P*rsmA* amplification (XbaI)	CCTTAC**TCTAGA**GTAACGAAAGATCTGACTGACCGC
P0777	*rsmB* deletion (XhoI)	GTC**CTCGAG**GCAGTAACAGTGTTTTGTTACC
P0778	*rsmB* deletion (XhoI)	AGT**CTCGAG**TGTAAGACAAGTCTCTCCCTC
P0782	*kan* amplification (XhoI)	AGT**CTCGAG**TTGTGTAGGCTGGAGCTGCTTC
P0783	*kan* amplification (XhoI)	GCG**CTCGAG**CCATATGAATATCCTCCTTAG
P0904	*expR1* deletion (XhoI)	TCAGTT**CTCGAG**TTATCCGACCGGTTCAGTCATAGGCT
P0917	+500 bp *rsmA* amplification (XbaI)	TCG**TCTAGA**GTCTCCGATAGGCATAAGGTGTG
P1028	P*pehA* amplification (XbaI)	CCTTAC**TCTAGA**AGCGCATCGAGGGAAGTATATAAC
P0914	*gacS* amplification (XbaI)	TCG**TCTAGA**GTCTCCGATAGGCATAAGGTGTG
P0915	*gacS* amplification (KpnI)	AGC**GGTACC**TGATCCGTCACGATCTGGACA
P1040	*expR1* amplification (SalI)	CCTTAC**GTCGAC**CATCTGTACAACTATCGTGGAAAGC
P1092	*gacS* deletion (XhoI)	CGAGCT**CTCGAG**TCCAATGTGGGAGAATTAGA
P1093	*gacS deletion (XhoI)*	CCTTAC**CTCGAG**TCAATGGCCGACATAGCCCG
P1094	*expR2* amplification (KpnI)	AGT**GGTACC**CAAAGAGCACGTAGCCGTCT
P1102	*expR1* deletion (XhoI)	GTG**CTCGAG**CTCATGACCTCTGCCTGAAA
P1103	*expR2* deletion (XhoI)	GTC**CTCGAG**GTAACGACCTCAATAAAAGC
P1104	*expR2* deletion (XhoI)	GTG**CTCGAG**CCATCATCACGTCTATTTCAC
P1106	*strep* amplification (XhoI)	AGC**CTCGAG**AGAGTCTTTGTTTTGACGCCAT

P1107	*strep* amplification (XhoI)	CTA**CTCGAG**ATCCTCTACGCCGGACGCATC
P1108	λ Red recombinase system amplification (SphI)	CCTTAC**GCATGC**CATCGATTTATTATGACAA
P1109	λ Red recombinase system amplification (XbaI)	CGAGCT**TCTAGA**TACCCATGGATTCTTCGTCT
P1211	*rsmB* amplification (PstI)	GTA**CTGCAG**AAGTTAGTAACCGGTTACAG
P1213	*rsmB* amplification (XmaI)	GTA**CCCGGG**GGAGAGACTTGTCTTACAGG
