# Supplementary material for: Signal Integration in Quorum Sensing Enables Cross-Species Induction of Virulence in Pectobacterium wasabiae
Source: mBio. 2017 May 23;8(3):e00398-17. doi: 10.1128/mBio.00398-17 (PMC5442451; doi:10.1128/mBio.00398-17)
Supplement: FIG S5 [file mbo003173315sf5.pdf]

TGTCCTGGAAGAATTCGGCACGAAAAAGCGTCAAATCTTAGCCCCGACGCTAGCAGCGTTCCTTT

CTGGCCTTAACCCGTTAGCAGTCTAATGGTTAATCGAATTTCACTTTCTGCTTTGTAAGAGATCTCTT

ACATTTGCTGTAAGAAATGTCTTATCTTTAATCTTGAAAATACGTTTCATTTTTATTTTTTCATTATTAA

TTAGCGAATTATAATAAAAAATAGTGGTATTGCTTAAGAGAGATTGAGAAACATTGTCACTTACCCC

lux box

TTGCCGAAAGCTGACAATTAATACATCTTATTACTT**AAGTTAGTAACCGGTTACAGTGTGTGTAAC**

-35 -10 **+1**

GGTGACTGTTGGTAACAAAACACTGTTACTGCACTCGGATGTCGACAGGCCTAGTTAAGGAGATATACATATGG

RsmA binding RBS GFP

CTAGCAAAGGAGAAGAACTCTTCACTGGAGTTGTCCCAATTCTTGTTGAATTAGATGGTGATGTTAACGGCCACAAGTTCTCTGTCAG

TGGAGAGGGTGAAGGTGATGCAACATACGGAAAACTTACCCTGAAGTTCATCTGCACTACTGGCAAACTGCCTGTTCCATGGCCAAC

ACTAGTCACTACTCTGTGCTATGGTGTTCAATGCTTTTCAAGATACCCGGATCATATGAAACGGCATGACTTTTTCAAGAGTGCCATGCC

CGAAGGTTATGTACAGGAAAGGACCATCTTCTTCAAAGATGACGGCAACTACAAGACACGTGCTGAAGTCAAGTTTGAAGGTGATAC

CCTTGTTAATAGAATCGAGTTAAAAGGTATTGACTTCAAGGAAGATGGCAACATTCTGGGACACAAATTGGAATACAACTATAACTCAC

ACAATGTATACATCATGGCAGACAAACAAAAGAATGGAATCAAAGTGAACTTCAAGACCCGCCACAACATTGAAGATGGAAGCGTTC

AACTAGCAGACCATTATCAACAAAATACTCCAATTGGCGATGGCCCTGTCCTTTTACCAGACAACCATTACCTGTCCACACAATCTGCCC

TTTCGAAAGATCCCAACGAAAAGAGAGACCACATGGTCCTTCTTGAGTTTGTAACAGCTGCTGGGATTACACATGGCATGGATGAACT

GTACAAC
