# Supplementary material for: Signal Integration in Quorum Sensing Enables Cross-Species Induction of Virulence in Pectobacterium wasabiae
Source: mBio. 2017 May 23;8(3):e00398-17. doi: 10.1128/mBio.00398-17 (PMC5442451; doi:10.1128/mBio.00398-17)
Supplement: TABLE S3 [file mbo003173315st3.pdf]

Table S3. Sequence of the *rsmA*^{sup} *expI* mutant. Strain SCC3193 and *rsmA*^{sup} *expI* mutant (RSV693) were sequenced by pair-end sequenced on an Illumina MiSeq Benchtop Sequencer. DNA library construction and sequencing was carried out by the IGC genomics facility. The mean coverage per sample was 39x and mutations present were identified using the BRESEQ pipeline (15) and manually inspected in IGV (16) using independent alignments performed with bwa mem (17). Mutations present in the *rsmA*^{sup} *expI* RSV693 strain but not in the SCC3193 are listed. We confirmed that the mutation L8L (TT<u>A</u>→TT<u>G</u>) identified in the *rpsA* gene of the *rsmA*^{sup} *expI* strain was not present in the ancestral SCC3193 strain nor in the *rsmA expI* parent strain (RSV531). Further work is necessary to demonstrate if this synonymous mutation is the mutation in the *rsmA*^{sup} *expI* mutant (RSV693) responsible for suppressing the growth defect observed in the parental *rsmA expI* mutant (RSV531).

**Position**	**Gene**	**Mutation**	**Description**
2119289	*rpsA*	L8L (TT<u>A</u>→TT<u>G</u>)	30S ribosomal protein S1
1112247 - 1112472	*rsmA*	Δ226 bp, streptomycin cassete insertion	Global regulator
4950126 - 4950209	*expI*	Δ100 Tn-10::cm insertion	Homoserine lactone synthetase
